# Targeted Screening for Alzheimer's Disease Clinical Trials Using Data-Driven Disease Progression Models

**DOI:** 10.3389/frai.2022.660581

**Published:** 2022-05-26

**Authors:** Neil P. Oxtoby, Cameron Shand, David M. Cash, Daniel C. Alexander, Frederik Barkhof

**Affiliations:** ^1^Centre for Medical Image Computing, Department of Computer Science, University College London, London, United Kingdom; ^2^Dementia Research Centre, Department of Neurodegenerative Disease, UCL Queen Square Institute of Neurology, University College London, London, United Kingdom; ^3^Amsterdam University Medical Center, Amsterdam, Netherlands

**Keywords:** disease progression modeling, Alzheimer's disease, mild cognitive impairment, clinical trials, screening, dementia, biomarkers, donepezil

## Abstract

Heterogeneity in Alzheimer's disease progression contributes to the ongoing failure to demonstrate efficacy of putative disease-modifying therapeutics that have been trialed over the past two decades. Any treatment effect present in a subgroup of trial participants (responders) can be diluted by non-responders who ideally should have been screened out of the trial. How to identify (screen-in) the most likely potential responders is an important question that is still without an answer. Here, we pilot a computational screening tool that leverages recent advances in data-driven disease progression modeling to improve stratification. This aims to increase the sensitivity to treatment effect by screening out non-responders, which will ultimately reduce the size, duration, and cost of a clinical trial. We demonstrate the concept of such a computational screening tool by retrospectively analyzing a completed double-blind clinical trial of donepezil in people with amnestic mild cognitive impairment (clinicaltrials.gov: NCT00000173), identifying a data-driven subgroup having more severe cognitive impairment who showed clearer treatment response than observed for the full cohort.

## 1. Introduction

Alzheimer's Disease (AD) is one of the most important socioeconomic challenges of the twenty-first century, being the leading cause of age-related dementia in an aging global population. Despite decades of research and clinical trials of potential therapies (Cummings et al., 2018b), no trials have been able to prove disease-modifying efficacy (Cummings et al., 2014, 2016, 2017, 2018a, 2019, 2020). There are multiple possible explanations for this. For example, potentially targeting the “wrong” pathology at the wrong time—typically amyloid protein pathogens are the target but if a treatment is given to symptomatic individuals, it may be too late to halt or reverse any damage done. Notwithstanding this, enrolling the right people at the right time (disease stage) into a clinical trial remains a considerable challenge because of undetected heterogeneity in phenotype/presentation (Firth et al., 2020) and/or ensuring the underlying pathology is present (Salloway et al., 2014), which can be a general problem because clinical trials often cannot adapt their designs to accommodate research discoveries made after they have begun. This can result in enrolment of non-responders into a clinical trial that wash out treatment effect in any subgroup of responders. Identification of non-responders typically occurs in *post hoc* subgroup analysis, which does not confer the benefits of a reduced trial size, and requires careful analysis to infer conclusions which can be misleading (Wang et al., 2007; Cummings, 2018). Given the breadth of evidence in support of the amyloid hypothesis (Hardy and Higgins, 1992) that has driven this clinical research for two decades, albeit with some controversies (Morris et al., 2014), here we focus on the aforementioned challenges of screening to identify the right participants at the right time. The good news is that there has been a swell of computational research into unraveling the heterogeneity of Alzheimer's disease progression over the past decade (e.g., see Oxtoby et al., 2017), driven largely by the increasing availability of large open medical datasets (Marinescu et al., 2018).

Computational approaches for aging and age-related diseases have been designed to fuse multimodal data into a quantitative template (Bilgel and Jedynak, 2019) of disease progression. These signatures often include a patient staging mechanism (Young et al., 2014) that provides a quantitative tool for fine-grained, individualized inference based on disease severity that goes above and beyond standard clinical phenotyping using patient symptoms. A recent innovation of data-driven disease progression modeling incorporates unsupervised machine learning, i.e., clustering, to provide both subtype and stage inference (Young et al., 2018). A frequent occurrence in this literature are claims of how these data-driven models can benefit clinical trials in Alzheimer's disease, but we are yet to find any evidence of studies actually analyzing clinical trial data to demonstrate the claimed benefit.

In this work we demonstrate the potential of data-driven models of disease progression to enhance clinical trials in Alzheimer's disease *via* targeted screening. We achieve this by example, using a particular modeling approach—the event-based model (Fonteijn et al., 2012)—in a *post hoc* subgroup analysis of a particular completed clinical trial that concluded without evidence of efficacy (Petersen et al., 2005).

## 2. Materials and Methods

This section describes the data, the computational model, and the statistical analysis used in our study. Overall, our analysis includes three steps. First, we fit a data-driven disease progression model of cognitive decline in AD to data from a large multicentre observational study, the Alzheimer's Disease Neuroimaging Initiative (ADNI; *training set*). Second, we use this computational model to score disease progression at baseline for participants in the completed “MCI” clinical trial from the Alzheimer's Disease Cooperative Study (ADCS-MCI; *test set*). Finally, this disease progression score is used to stratify the ADCS-MCI Trial participants for a *post hoc* analysis of subgroup treatment effect.

### 2.1. Data

**Figure 1:**
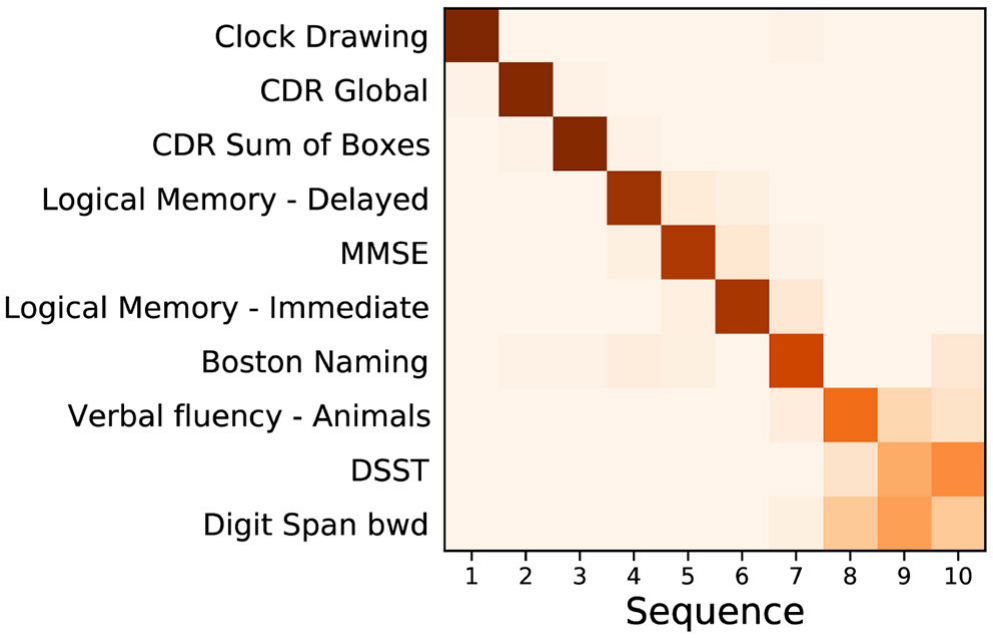
Event based model of cognitive decline (ADNI). Positional density/variance diagram showing the sequence (top to bottom) and uncertainty (left to right) under five-fold cross-validation (repeated 10 times). CDR, clinical dementia rating; MMSE, mini-mental state examination; bwd, backward; DSST, digit symbol substitution test.

Our reference model fit to data from the ADNI observational study is used to stage participants from the ADCS-MCI clinical trial (clinicaltrials.gov: NCT00000173; Petersen et al., 2005). For this we use a set of features common to both data sets, which is a subset of cognitive instruments used in the ADCS-MCI trial (see the vertical axis of Results, Figure 1), taking care to exclude ADAS-Cog (being a secondary outcome of the trial).^1^ For simplicity, we included only ADNI participants having complete data for this feature set.

Data used in the preparation of this article were obtained from the Alzheimer's Disease Neuroimaging Initiative (ADNI) database (adni.loni.usc.edu). The ADNI was launched in 2003 as a public-private partnership, led by Principal Investigator Michael W. Weiner, MD. The primary goal of ADNI has been to test whether serial magnetic resonance imaging (MRI), positron emission tomography (PET), other biological markers, and clinical and neuropsychological assessment can be combined to measure the progression of mild cognitive impairment (MCI) and early Alzheimer's disease (AD).

Additional data used in the preparation of this article were obtained from the Alzheimer's Disease Cooperative Study (ADCS) database (adcs.org). Specifically, we analyse data from the completed ADCS-MCI clinical trial of donepezil and vitamin E, reported in Petersen et al. (2005). The ADCS-MCI trial aimed to assess the efficacy of vitamin E and donepezil in subjects with amnestic MCI. The primary end point was the time to the development of possible or probable AD dementia, with secondary outcomes on cognition and function. Measurements were taken at 6-month intervals until the end of the trial (36 months). At screening, 769 subjects were included in the trial, randomized into 259, 257, and 253 subjects for the placebo, vitamin E, and donepezil arms, respectively—reducing to 174, 158, and 145 by the end of the trial.

### 2.2. Event-Based Model

The event-based model (EBM) (Fonteijn et al., 2012; Young et al., 2014) estimates the most likely sequence, and uncertainty in this sequence, of observable cumulative abnormality events in the pathophysiological cascade (Jack et al., 2010) of a progressive disease. In this context, an event constitutes deviation of a biomarker measurement from those typical of healthy controls, toward those typical of patients. Events, and the overall sequence of events, are probabilistic entities. The EBM sequence of cumulative abnormality is estimated from cross-sectional data. This is made possible by combining data from a cohort of individuals at different stages of cumulative abnormality. The EBM sequence estimation is achieved directly from the data distributions in diseased and healthy groups and without *a priori*-defined disease stages or biomarker cutpoints /thresholds. The EBM, in its various versions, has been applied to a variety of diseases since 2011 (e.g., Fonteijn et al., 2012; Eshaghi et al., 2018; Oxtoby et al., 2018, 2021; Wijeratne et al., 2018; Firth et al., 2020). For a detailed intuitive description of the EBM, we refer the reader to Oxtoby et al. (2021).

Here, we employ the recently-developed kernel density estimation (KDE) EBM that copes naturally with the ceiling/floor effects seen in cognitive data (Firth et al., 2020), and gives a cleaner interpretation of the model by exploiting prior information on disease direction (Oxtoby et al., 2021). To improve generalizability, we perform repeated five-fold cross-validation (10 repeats) and combine all 50 sets of posterior samples of the EBM into a cross-validated positional density map (Oxtoby et al., 2021).

The EBM affords us a screening tool by way of the patient staging mechanism introduced by Young et al. (2014). This process assigns a model stage (disease progression score) that maximizes the likelihood given an individual's set of measurements. Here, we use the ADNI-trained EBM to stage baseline data from the ADCS-MCI clinical trial, then stratify subjects into strata based on disease progression scores for *post hoc* subgroup analyses. In future, this process could be performed as part of the screening process to homogenize the clinical trial cohort.

### 2.3. Statistical Analysis

Our hypothesis is that AD clinical trial cohorts are likely to contain undetected heterogeneity that washes out treatment effects which may exist in an independently identifiable subgroup of responders. Accordingly, in order to examine whether our proposed screening tool can detect this heterogeneity and reveal such a subgroup of responders, our *post hoc* subgroup analysis of the ADCS-MCI clinical trial closely follows the primary analyses in Petersen et al. (2005). We describe the key steps below.

**Primary Outcome:** We use Kaplan–Meier estimators to estimate the rate of progression from MCI to AD over the course of the trial. Additionally, Cox proportional-hazards models were constructed to compare the risk for progression in each treatment arm with the placebo (using baseline age, MMSE, and APOE-ϵ4 carrier status as covariates). This intention-to-treat analysis in the trial was conducted for both placebo vs. vitamin E and placebo vs. donepezil, but in this paper we focus on the latter.

To correct for multiple comparisons in the Cox proportional-hazards model (for the two treatment arms), the Hochberg method was used. As our introduction of subgroups increases the number of comparisons made, we extend this adjustment for the total number of subgroups, regardless of whether a single subgroup is the focus of analysis.

**Secondary Outcome:** We compare ADAS-Cog 13 scores between placebo and donepezil arms in subgroups at each 6-month interval to assess the difference in longitudinal cognitive decline. A two-sided Mann–Whitney *U*-test is used to compare the treatment groups at each time point for each subgroup, correcting for multiple comparisons using the Hochberg method.

## 3. Results

### 3.1. Reference Model

Figure 1 shows a positional variance diagram for an event-based model (Firth et al., 2020) of cognitive decline due to probable Alzheimer's disease, across a set of cognitive instruments from *N* = 810 (of 2,040) ADNI participants [229 cognitively normal (CN), 181 AD, 400 MCI] having complete data (see Section 2). The cross-validated model's confidence in the sequence is higher where the positional variance is reduced—a dark diagonal corresponds to strong confidence in the data-driven ordering. The estimated sequence of cognitive decline starts from the Clock Drawing test and Clinical Dementia Rating (CDR), through tests of memory recall (Logical Memory) and general cognition (MMSE), to verbal fluency (Boston Naming; Animals), working memory (Digit span backwards), and executive function (Digit Symbol Substitution Test, DSST).

**Figure 2:**
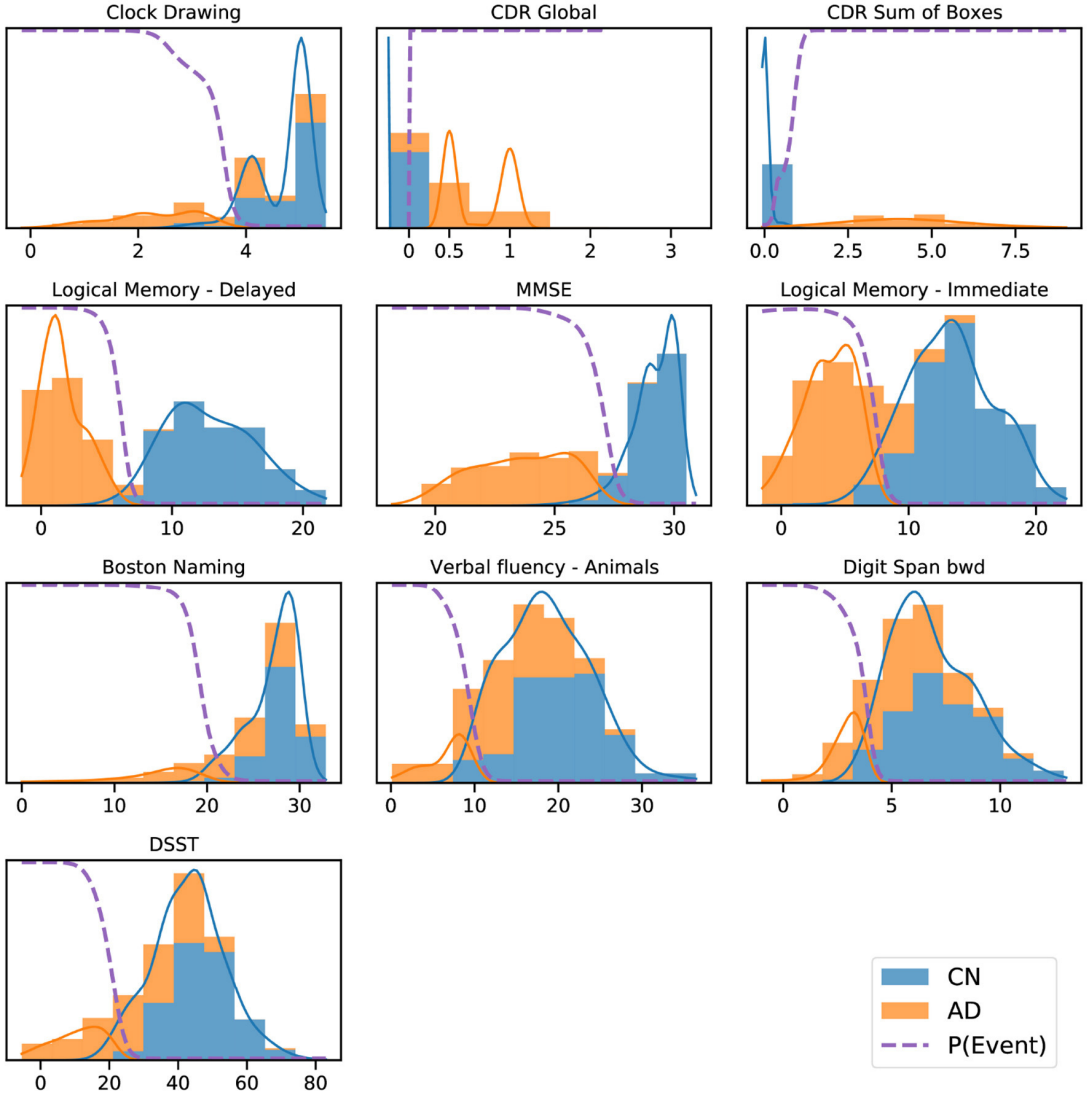
ADNI data histograms (adjusted for age and education level) and EBM mixture models for each feature. Orange bars corresponds to AD patient data, blue bars to data from CN participants, showing the “normal” and “abnormal” distributions and the determined probability of the event having occurred (dashed line).

Figure 2 shows a key component of the EBM—the normal/abnormal mixture models for each cognitive instrument (blue/orange solid lines, respectively), and the resulting cumulative probability of an event having occurred (dashed lines) (Fonteijn et al., 2012). These sigmoidal event probabilities quantify divergence from normality (Oxtoby et al., 2021) and provide a visualization of the data-driven event threshold (akin to a data-driven biomarker cutpoint). Histograms show the AD (orange) and CN (blue) data from ADNI. Early/late events are, respectively, those that have occurred in many/few patients and thus show greater/smaller separation between the group histograms.

### 3.2. Patient Staging: Re-screening the ADCS-MCI Trial

**Figure 3:**
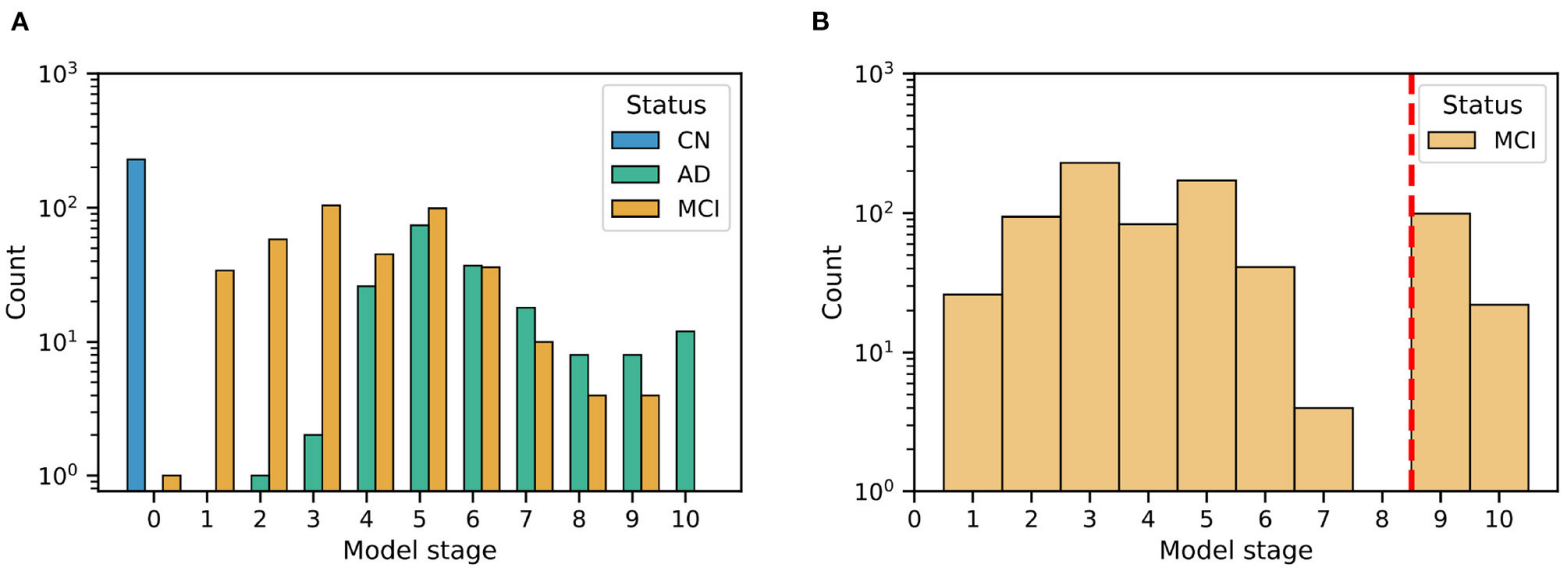
Histograms of model stage for subjects in the ADNI dataset **(A)** and ADCS-MCI trial **(B)**.

Figure 3 shows the distribution of patient stages assigned to participants in the (Figure 3A) ADNI study and (Figure 3B) ADCS-MCI trial, using the ADNI-trained EBM shown in Figure 3. The MCI distributions show considerable heterogeneity, with a notable late-stage ADCS-MCI subgroup beyond stage 8 in Figure 3B, delineated by a red dashed line. Table 1 compares the whole ADCS-MCI cohort and 2 subgroups (“Late-stage” and “Others”) on demographic and cognitive measures at baseline.

**Table 1:** Demographic and cognitive comparison of ADCS-MCI trial participants (All) and the model-determined subgroups thereof (“Late-stage” and “Others”).

	**Group**		
	**All**	**Others**	**Late-stage**
**Measure**	**(N = 769)**	**(N = 648)**	**(N = 121)**
Age (years)	72.9 (7.3)	73.0 (7.2)	72.4 (7.9)
Education (years)	14.6 (3.1)	14.6 (3.1)	15.0 (3.0)
Sex (% female)	352 (45.8%)	290 (44.8%)	62 (51.2%)
APOE-ϵ4 carrier (%)	424 (55.1%)	352 (54.3%)	72 (59.5%)
Donepezil arm (%)	253 (32.9%)	219 (33.8%)	34 (28.1%)
Vitamin E arm (%)	257 (33.4%)	216 (33.3%)	41 (33.9%)
Placebo arm (%)	259 (33.7%)	213 (32.9%)	46 (38.0%)
ADAS-Cog 11	11.3 (4.4)	10.8 (4.2)	14.1 (4.0)
ADAS-Cog 13	17.7 (6.1)	17.0 (5.9)	21.6 (5.6)
ADAS-Cog Q4	6.3 (2.2)	6.1 (2.2)	7.3 (2.0)
Boston naming	6.9 (2.4)	7.3 (2.2)	5.1 (2.5)
CDR global	0.5 (0.0)	0.5 (0.0)	0.5 (0.0)
CDR sum of boxes	1.8 (0.8)	1.8 (0.8)	2.2 (0.8)
Clock drawing	4.3 (0.9)	4.5 (0.8)	3.4 (1.0)
Digit span bwd	6.2 (2.1)	6.4 (2.1)	5.1 (1.9)
DSST	31.5 (10.9)	33.4 (10.2)	21.1 (8.0)
Logical memory - delayed	3.3 (2.4)	3.5 (2.5)	2.2 (2.0)
Logical memory - immediate	6.2 (3.1)	6.5 (3.1)	4.7 (2.7)
MMSE	27.3 (1.8)	27.5 (1.8)	26.2 (1.7)
Verbal fluency - animals	15.8 (5.2)	16.8 (5.0)	10.5 (3.0)

**Figure 4:**
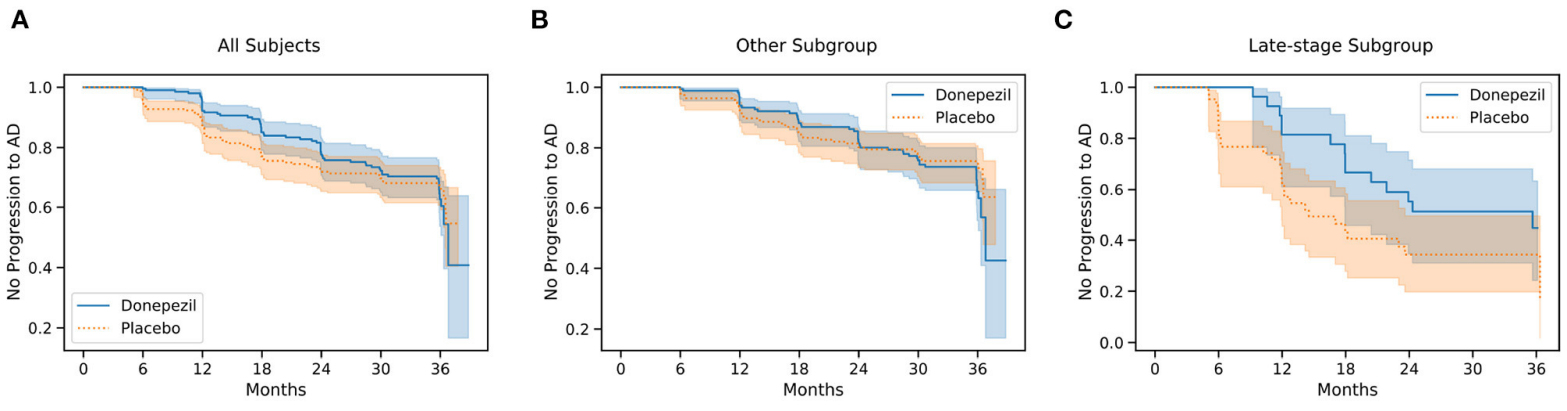
Kaplan–Meier survival curves for all 769 participants **(A)**, the “Others” subgroup **(B)**, and the “Late-stage” subgroup **(C)** in the ADCS-MCI trial.

**Figure 5:**
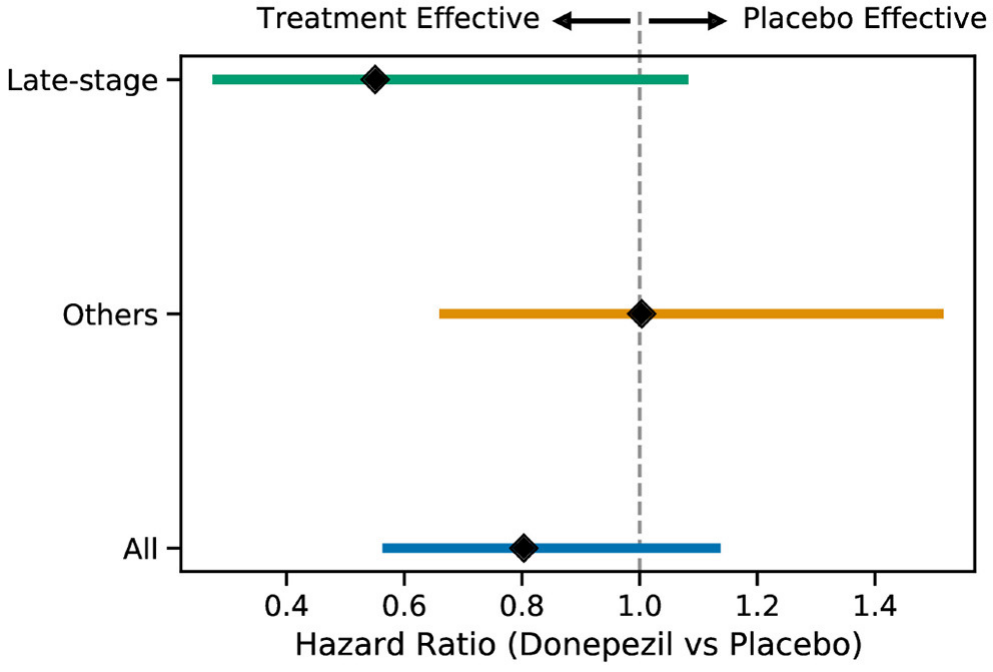
Hazard ratios (with 95% confidence intervals) for the progression to AD for the two subgroups and all subjects when comparing the placebo and donepezil arms.

**Primary Outcome:** Figure 4 shows Kaplan–Meier curves for the whole ADCS-MCI cohort (Figure 4A), the early-to-middle “Others” subgroup (Figure 4B) and the “Late-stage” subgroup (Figure 4C) in the placebo and donepezil arms, illustrating the change in survival rates (specifically, not progressing to probable AD dementia) during the trial. For each survival function estimate, 95% confidence intervals are shown in the shaded area. Figure 5 shows the corresponding hazard ratios and 95% confidence intervals for Cox proportional-hazards models quantifying the risk of progression from MCI to AD. Although there are no significant differences between all subjects (hazard ratio 0.80; 95% CI 0.57–1.13; *p* = 0.42), the estimated effect seems larger than in the early-to-middle stage subgroup (hazard ratio 1.00; 95% CI 0.67–1.51; *p* = 0.99), or the late-stage subgroup (hazard ratio 0.55; 95% CI 0.28–1.07; *p* = 0.24).

**Figure 6:**
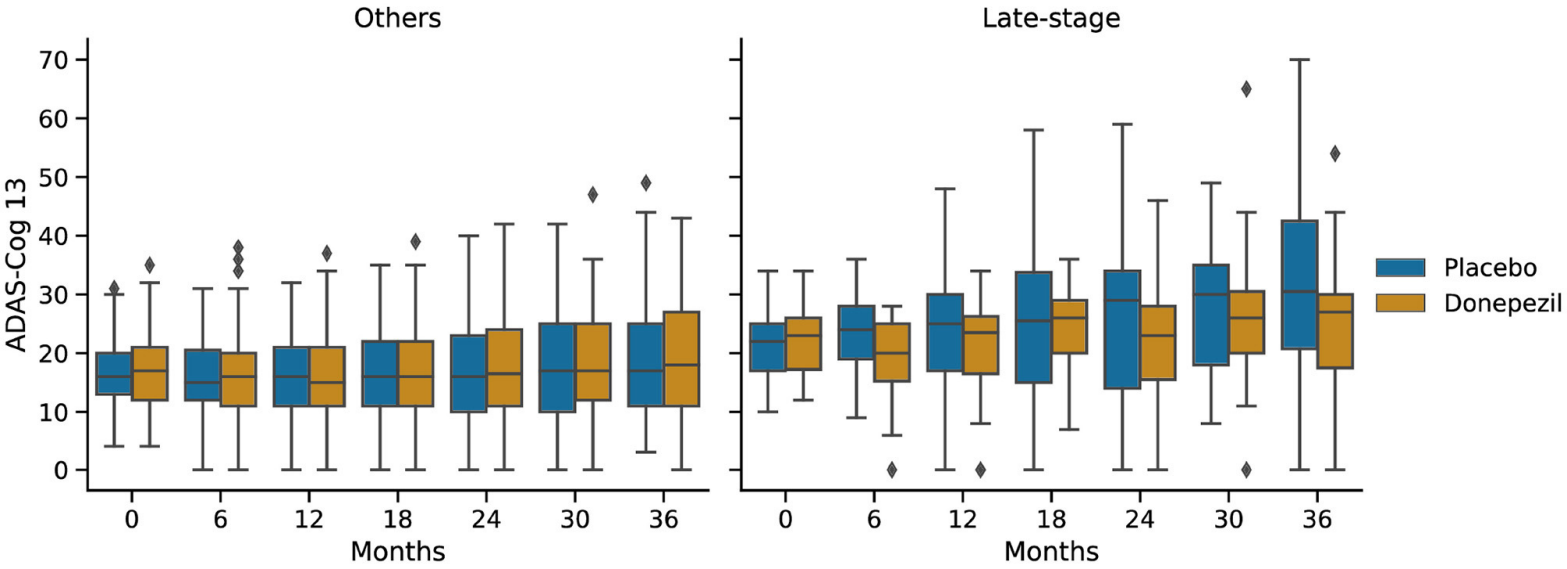
Progression of ADAS-Cog 13 scores in the placebo and donepezil arms throughout the trial for each of the two subgroups.

Figure 6 shows ADAS-Cog 13 scores at 6-month intervals throughout the ADCS-MCI trial separately for the two subgroups. Conducting a two-sided Mann–Whitney *U*-test at each time point, no significant difference (in adjusted *p*-values) was found in either subgroup, despite the apparent trend toward treatment effect in the late-stage subgroup.

## 4. Discussion

We fit an event-based model of cognitive decline in Alzheimer's disease using a reference data set (ADNI), which was then used to score disease progression in subjects at baseline in a completed clinical trial (ADCS-MCI). This disease progression score was used to stratify trial participants for a *post hoc* subgroup analysis of treatment effect.

The event-based model of cognitive decline in Figure 1 is representative of typical (memory-led) Alzheimer's disease, with CDR and impaired memory recall occurring before decline in verbal fluency, working memory, and executive function. Indeed, the estimated sequence shares similarities with results in Firth et al. (2020), which involved an independent cohort. We deliberately excluded ADAS-Cog scores from the model to avoid circularity with the corresponding secondary outcome of the trial (and also to avoid having to perform the relatively arduous ADAS-Cog test at a screening visit). Supplementary Figure 1 shows that the sequence is largely unchanged with ADAS-Cog features included. Notably, Clock Drawing appears as the first event (before even CDR features), albeit with an additional component of positional density around stages 7–9, supporting the presence of additional heterogeneity among individuals. This result warrants further investigation but is beyond the scope of our study.

The event-based model patient staging mechanism (Young et al., 2014) revealed considerable heterogeneity in the cognitive impairment of MCI participants in both the ADNI observational study (Figure 3A) and the ADCS-MCI clinical trial (Figure 3B). Such clinical heterogeneity is likely to mask treatment response in clinical trials, particularly if the underlying source is biological heterogeneity relevant to the experimental treatment. The biological underpinnings here are unknown due to the absence of biomarker data in the ADCS-MCI trial, and we need access to such individual-level biomarker data from more recent clinical trials if we are to assess the value of EBM screening vs. biomarker screening. Regardless, we found promising trends in our *post hoc* subgroup analyses (discussed below). Of course, the reduced sample size increases screen-in cost of a clinical trial and potentially diminishes the treatable patient group (affecting also the drug label). This is mostly positive. Pros: a medicine that is effective on a subgroup is better than no medicine at all; not treating non-responders reduces the occurrence of unnecessary side-effects. Con: the smaller group of potential responders limits the treatable patient population (but at least those treated are likely to benefit).

In the ADCS-MCI trial we found encouraging trends toward improved survival (Figure 4), preserved cognition (Figure 6), and a lower hazard ratio (Figure 5) in the more severely affected “Late-stage” MCI subgroup (*N* = 121) compared to the less affected “Others” subgroup (*N* = 648). These results suggest that the treatment (donepezil) may protect cognition and provide more protection against MCI conversion to dementia for late-stage MCI. This result concurs with the fact that donepezil is approved for symptomatic relief in more severely affected groups—specifically, dementia patients. Additionally, a recent re-analysis of the ADCS-MCI trial unmasked beneficial effects of donepezil (Edmonds et al., 2018) in a more severely affected subgroup by screening out false-positive MCI participants using hierarchical clustering by Ward's method.

There are multiple possible explanations for why more severely impaired individuals with MCI seem to benefit from donepezil preferentially over less impaired individuals. For one, donepezil may have less cognitive benefit earlier in the disease. Another is that ADAS-Cog might be inadequate to detect such a benefit. Regardless, the key finding is that our approach was able to stratify a clinical trial population into potential responders and non-responders using only baseline/screening data. This supports the notion that computational, data-driven screening can substantially reduce the size (and cost) of a clinical trial, without sacrificing statistical power (see also Franzmeier et al., 2020).

Our work motivates using event-based model staging as a screening tool to enrich clinical trials, but the general principle can be applied using other models that can calculate disease progression scores (e.g., Jedynak et al., 2012; Leoutsakos et al., 2016; Stallard et al., 2017; Wang et al., 2020). While many such works mention the potential application to analyzing clinical trial data, fewer suggest incorporating this into the screening stage, and none (to our knowledge) have actually applied such models in clinical trials, nor in *post hoc* analyses that follow the original analysis protocol to retrospectively determine subgroup treatment effects. Closest to this work is the aforementioned study of the ADCS-MCI trial data by Edmonds et al. (2018), and the work of Schneider et al. (2016), but the approaches used in these studies do not provide an interpretable disease progression signature, nor do they allow for future extension to seamlessly incorporate imaging data and other biomarkers.

In summary, the ADCS-MCI trial was an attempt to test whether donepezil, an approved symptomatic treatment of dementia patients, could slow progression from MCI to dementia. This placebo-controlled, double-blind, phase 3 study found no significant treatment effects (Petersen et al., 2005). Here, we reanalyzed the trial in a *post hoc* subgroup analysis, with the subgroups defined by a data-driven disease progression model: the event-based model (Fonteijn et al., 2012; Young et al., 2014; Firth et al., 2020). Our two key findings are: (1) there was considerable heterogeneity in cognitive impairment in the ADCS-MCI trial, suggesting an inadequate screening protocol; (2) this heterogeneity masked a possible treatment effect in a sample of more severely impaired late-stage MCI participants, despite the likelihood of this smaller sample being under-powered to detect an effect of this magnitude. Our study has highlighted a potential mechanism for improving clinical trial design but the general applicability will require broader verification, ideally in more recent trials having biomarker data.

In conclusion, our findings support the use of our proposed data-driven screening method to enhance targeting and efficiency of future clinical trials in Alzheimer's disease. What is perhaps most exciting in the immediate future is the prospect of performing similar *post hoc* analyses in other “failed” clinical trials, which could resurrect some Alzheimer's disease drug research programs, saving billions of dollars and years of research. This work is continuing.

## Author's Note

Data used in preparation of this article were obtained from the Alzheimer's Disease Neuroimaging Initiative (ADNI) database (adni.loni.usc.edu). As such, the investigators within the ADNI contributed to the design and implementation of ADNI and/or provided data but did not participate in analysis or writing of this report. A complete listing of ADNI investigators can be found at: http://adni.loni.usc.edu/wp-content/uploads/how_to_apply/ADNI_Acknowledgement_List.pdf.

Data used in the preparation of this manuscript were obtained from the Alzheimer's Disease Cooperative Study legacy database.

## Data Availability Statement

The original contributions presented in the study are included in the article/Supplementary Material, further inquiries can be directed to the corresponding author.

## Ethics Statement

Ethical review and approval was not required for the study on human participants in accordance with the local legislation and institutional requirements. The patients/participants provided their written informed consent to participate in this study.

## Author Contributions

NO conceived of the study and obtained funding. NO and CS performed the data analysis and drafted the manuscript. All authors contributed to interpretation of results and manuscript writing. All authors contributed to the article and approved the submitted version.

## Funding

NO is a UKRI Future Leaders Fellow. NO and CS acknowledge funding from the UKRI Medical Research Council (MRC MR/S03546X/1). NO, DA, and FB acknowledge funding from the EuroPOND project. This project has received funding from the European Union's Horizon 2020 Research and Innovation Programme under grant agreement no. 666992—and the National Institute for Health Research University College London Hospitals Biomedical Research Centre.

## Conflict of Interest

The authors declare that the research was conducted in the absence of any commercial or financial relationships that could be construed as a potential conflict of interest.

## Publisher's Note

All claims expressed in this article are solely those of the authors and do not necessarily represent those of their affiliated organizations, or those of the publisher, the editors and the reviewers. Any product that may be evaluated in this article, or claim that may be made by its manufacturer, is not guaranteed or endorsed by the publisher.

## Footnotes

^1^Results with ADAS-Cog included can be found in the Supplementary Material.

## References

[B1] Bilgel M. Jedynak B. M. (2019). Predicting time to dementia using a quantitative template of disease progression. Alzheimers Dement. 11, 205–215. 10.1016/j.dadm.2019.01.005 30859120 PMC6396328

[B2] Cummings J. (2018). Lessons learned from Alzheimer disease: clinical trials with negative outcomes. Clin. Transl. Sci. 11, 147–152. 10.1111/cts.12491 28767185 PMC5866992

[B3] Cummings J. Lee G. Mortsdorf T. Ritter A. Zhong K. (2017). Alzheimer's disease drug development pipeline: 2017. Alzheimers Dement. 3, 367–384. 10.1016/j.trci.2017.05.002 29067343 PMC5651419

[B4] Cummings J. Lee G. Ritter A. Sabbagh M. Zhong K. (2019). Alzheimer's disease drug development pipeline: 2019. Alzheimers Dement. 5, 272–293. 10.1016/j.trci.2019.05.008 31334330 PMC6617248

[B5] Cummings J. Lee G. Ritter A. Sabbagh M. Zhong K. (2020). Alzheimer's disease drug development pipeline: 2020. Alzheimers Dement. 6, e12050. 10.1002/trc2.12050 32695874 PMC7364858

[B6] Cummings J. Lee G. Ritter A. Zhong K. (2018a). Alzheimer's disease drug development pipeline: 2018. Alzheimers Dement. 4, 195–214. 10.1016/j.trci.2018.03.009 29955663 PMC6021548

[B7] Cummings J. Morstorf T. Lee G. (2016). Alzheimer's drug-development pipeline: 2016. Alzheimers Dement. 2, 222–232. 10.1016/j.trci.2016.07.001 29067309 PMC5651348

[B8] Cummings J. Ritter A. Zhong K. (2018b). Clinical trials for disease-modifying therapies in Alzheimer's disease: a primer, lessons learned, and a blueprint for the future. J. Alzheimers Dis. 64, S3–S22. 10.3233/JAD-179901 29562511 PMC6004914

[B9] Cummings J. L. Morstorf T. Zhong K. (2014). Alzheimer's disease drug-development pipeline: few candidates, frequent failures. Alzheimers Res. Therapy 6, 37. 10.1186/alzrt269 25024750 PMC4095696

[B10] Edmonds E. C. Ard M. C. Edland S. D. Galasko D. R. Salmon D. P. Bondi M. W. (2018). Unmasking the benefits of donepezil via psychometrically precise identification of mild cognitive impairment: a secondary analysis of the ADCS vitamin e and donepezil in MCI study. Alzheimers Dement. 4, 11–18. 10.1016/j.trci.2017.11.001 29296659 PMC5738722

[B11] Eshaghi A. Marinescu R. V. Young A. L. Firth N. C. Prados F. Jorge Cardoso M.. (2018). Progression of regional grey matter atrophy in multiple sclerosis. Brain 141, 1665–1677. 10.1093/brain/awy088 29741648 PMC5995197

[B12] Firth N. C. Primativo S. Brotherhood E. Young A. L. Yong K. X. Crutch S. J.. (2020). Sequences of cognitive decline in typical Alzheimer's disease and posterior cortical atrophy estimated using a novel event-based model of disease progression. Alzheimers Dement. 16, 965–973. 10.1002/alz.12083 32489019 PMC8432168

[B13] Fonteijn H. M. Modat M. Clarkson M. J. Barnes J. Lehmann M. Hobbs N. Z.. (2012). An event-based model for disease progression and its application in familial Alzheimer's disease and Huntington's disease. Neuroimage 60, 1880–1889. 10.1016/j.neuroimage.2012.01.062 22281676

[B14] Franzmeier N. Koutsouleris N. Benzinger T. Goate A. Karch C. M. Fagan A. M.. (2020). Predicting sporadic Alzheimer's disease progression via inherited Alzheimer's disease-informed machine-learning. Alzheimers Dement. 16, 501–511. 10.1002/alz.12032 32043733 PMC7222030

[B15] Hardy J. Higgins G. (1992). Alzheimer's disease: the amyloid cascade hypothesis. Science 256, 184–185. 10.1126/science.1566067 1566067

[B16] Jack C. R. Knopman D. S. Jagust W. J. Shaw L. M. Aisen P. S. Weiner M. W.. (2010). Hypothetical model of dynamic biomarkers of the Alzheimer's pathological cascade. Lancet Neurol. 9, 119–128. 10.1016/S1474-4422(09)70299-6 20083042 PMC2819840

[B17] Jedynak B. M. Lang A. Liu B. Katz E. Zhang Y. Wyman B. T.. (2012). A computational neurodegenerative disease progression score: method and results with the Alzheimer's disease neuroimaging initiative cohort. Neuroimage 63, 1478–1486. 10.1016/j.neuroimage.2012.07.059 22885136 PMC3472161

[B18] Leoutsakos J.-M. Gross A. Jones R. Albert M. Breitner J. (2016). “Alzheimer's progression score”: development of a biomarker summary outcome for ad prevention trials. J. Prev. Alzheimers Dis. 3, 229. 10.14283/jpad.2016.120 29034223 PMC5639716

[B19] Marinescu R. V. Oxtoby N. P. Young A. L. Bron E. E. Toga A. W. Weiner M. W.. (2018). TADPOLE challenge: prediction of longitudinal evolution in Alzheimer's disease. arXiv preprint arXiv:1805.03909. 10.48550/arXiv.1805.03909

[B20] Morris G. P. Clark I. A. Vissel B. (2014). Inconsistencies and controversies surrounding the amyloid hypothesis of Alzheimer's disease. Acta Neuropathol. Commun. 2, 135. 10.1186/PREACCEPT-1342777270140958 25231068 PMC4207354

[B21] Oxtoby N. Leyland L. Aksman L. Thomas G. Bunting E. Wijeratne P.. (2021). Sequence of clinical and neurodegeneration events in parkinson's disease progression. Brain J. Neurol. 144, 975–988. 10.1093/brain/awaa461 33543247 PMC8041043

[B22] Oxtoby N. P. Alexander D. C. EuroPOND Consortium (2017). Imaging plus X: multimodal models of neurodegenerative disease. Curr. Opin. Neurol. 30, 371–379. 10.1097/WCO.0000000000000460 28520598 PMC5491241

[B23] Oxtoby N. P. Young A. L. Cash D. M. Benzinger T. L. S. Fagan A. M. Morris J. C.. (2018). Data-driven models of dominantly-inherited Alzheimer's disease progression. Brain 141, 1529–1544. 10.1093/brain/awy050 29579160 PMC5920320

[B24] Petersen R. C. Thomas R. G. Grundman M. Bennett D. Doody R. Ferris S.. (2005). Vitamin E and donepezil for the treatment of mild cognitive impairment. N. Engl. J. Med. 352, 2379–2388. 10.1056/NEJMoa050151 15829527

[B25] Salloway S. Sperling R. Fox N. C. Blennow K. Klunk W. Raskind M.. (2014). Two phase 3 trials of bapineuzumab in mild-to-moderate Alzheimer's disease. N. Engl. J. Med. 370, 322–333. 10.1056/NEJMoa1304839 24450891 PMC4159618

[B26] Schneider L. S. Frangakis C. Drye L. T. Devanand D. Marano C. M. Mintzer J.. (2016). Heterogeneity of treatment response to citalopram for patients with Alzheimer's disease with aggression or agitation: the citad randomized clinical trial. Am. J. Psychiatry 173, 465–472. 10.1176/appi.ajp.2015.15050648 26771737 PMC6419726

[B27] Stallard E. Kinosian B. Stern Y. (2017). Personalized predictive modeling for patients with Alzheimer's disease using an extension of Sullivan's life table model. Alzheimers Res. Therapy 9, 75. 10.1186/s13195-017-0302-6 28931428 PMC5607490

[B28] Wang R. Lagakos S. W. Ware J. H. Hunter D. J. Drazen J. M. (2007). Statistics in medicine–reporting of subgroup analyses in clinical trials. N. Engl. J. Med. 357, 2189–2194. 10.1056/NEJMsr077003 18032770

[B29] Wang Z. Tang Z. Zhu Y. Pettigrew C. Soldan A. Gross A.. (2020). Ad risk score for the early phases of disease based on unsupervised machine learning. Alzheimers Dement. 16, 1524–1533. 10.1002/alz.12140 32729964 PMC7666001

[B30] Wijeratne P. A. Young A. L. Oxtoby N. P. Marinescu R. V. Firth N. C. Johnson E. B.. (2018). An image-based model of brain volume biomarker changes in huntington's disease. Ann. Clin. Transl. Neurol. 5, 570–582. 10.1002/acn3.558 29761120 PMC5945962

[B31] Young A. L. Marinescu R. V. Oxtoby N. P. Bocchetta M. Yong K. Firth N. C.. (2018). Uncovering the heterogeneity and temporal complexity of neurodegenerative diseases with Subtype and Stage Inference. Nat. Commun. 9, 4273. 10.1038/s41467-018-05892-0 30323170 PMC6189176

[B32] Young A. L. Oxtoby N. P. Daga P. Cash D. M. Fox N. C. Ourselin S.. (2014). A data-driven model of biomarker changes in sporadic Alzheimers disease. Brain 137, 2564–2577. 10.1093/brain/awu176 25012224 PMC4132648

