# Supplementary material for: Targeted Screening for Alzheimer's Disease Clinical Trials Using Data-Driven Disease Progression Models
Source: Front Artif Intell. 2022 May 26;5:660581. doi: 10.3389/frai.2022.660581 (PMC9204250; doi:10.3389/frai.2022.660581)
Supplement: Supplementary file 1 [file Data_Sheet_1.pdf]

frontiers

Supplementary Material: Targeted screening for Alzheimer's disease clinical trials using data-driven disease progression models

Neil P. Oxtoby, *et al.*

1 EVENT-BASED MODEL WITH ADAS-COG

In the main paper we excluded ADAS-Cog from the cognitive instruments used to construct the reference model, allowing separate secondary validation of the subgroups. Here, we provide results with ADAS-Cog included.

1.1 Reference Model

Figure S1 shows the positional variance diagram for the $N = 803$ (of 2040) ADNI participants (229 CN, 177 AD, 397 MCI) with complete data. Compared to Figure 1 in the main paper, the ADAS-Cog features lie grouped together in the middle of the sequence (with the uncertainty around their relative ordering likely driven by their high correlation). The overall sequence is otherwise preserved aside from a swap of the neighbouring DSST and digit span backwards, supporting the robustness of the sequence.

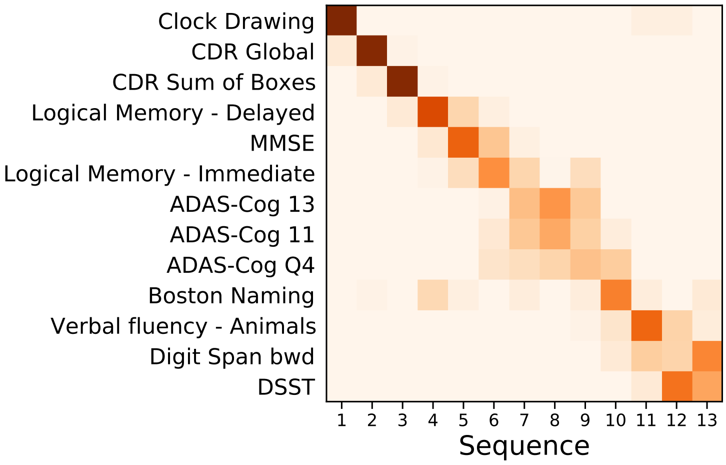

Figure S1. Event based model of cognitive decline (ADNI). Positional density/variance diagram showing the sequence (top to bottom) and uncertainty (left to right) under 5-fold cross-validation (repeated 10 times). Abbreviations: ADAS-Cog — Alzheimer's Disease Assessment Scale-Cognitive Subscale; CDR — clinical dementia rating; MMSE — mini-mental state examination; bwd — backward; DSST — digit symbol substitution test.

The mixture models corresponding to Figure S1 are shown in Figure S2, which includes ADAS-Cog, highlighting the generally higher scores for AD subjects over those that are cognitively normal. As the mixture models are fit independently for each biomarker, the others are identical to those in the main paper, highlighting the flexibility of this approach to take advantage of additional data where available.

1.2 Patient staging

The model stage distribution for ADCS-MCI trial participants is shown in Figure S3, where (in contrast with the main paper) there are three identifiable subgroups (consisting of 648, 121, and 92 subjects).

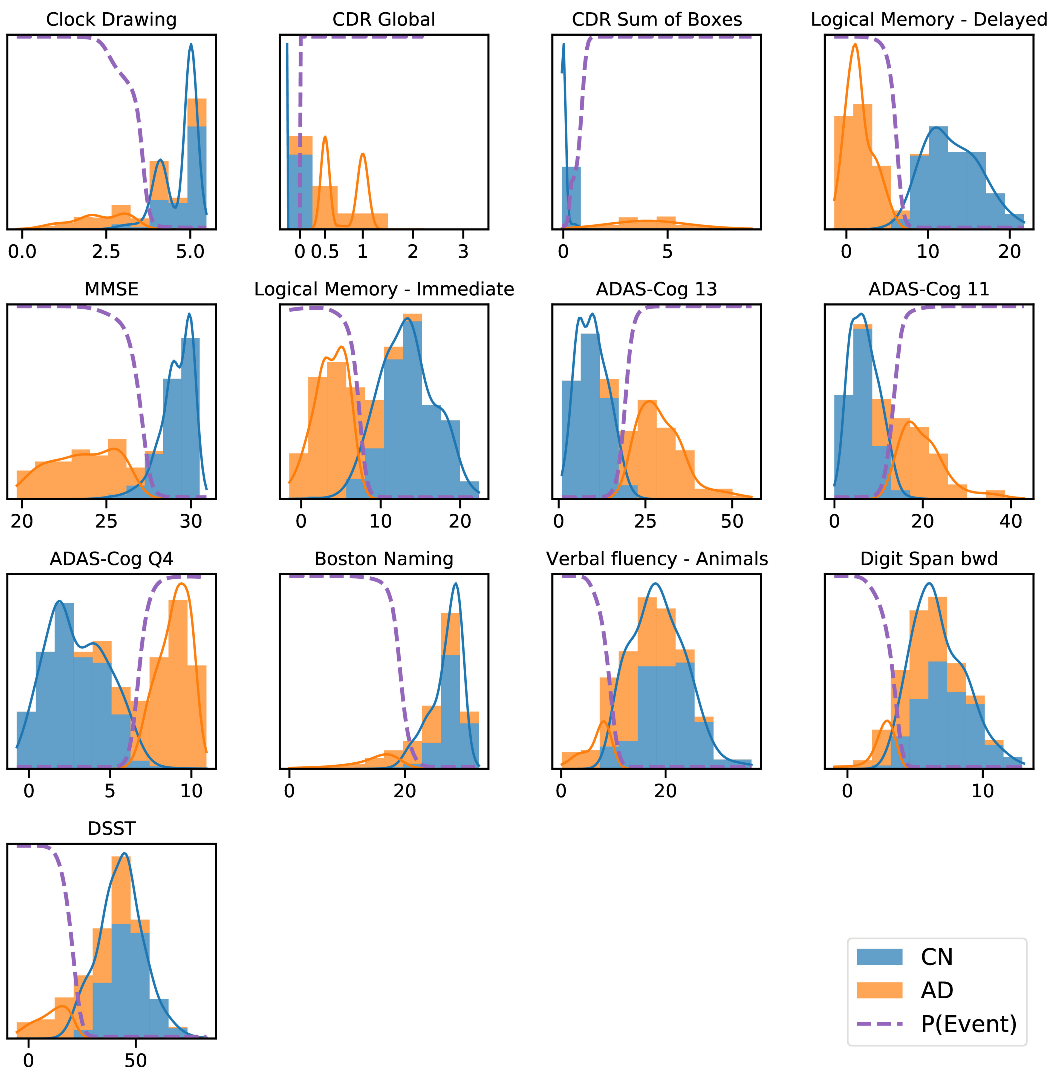

Figure S2. ADNI data histograms (adjusted for age and education level) and EBM mixture models for each feature. Orange bars corresponds to AD patient data, blue bars to data from CN participants, showing the “normal” and “abnormal” distributions and the determined probability of the event having occurred (dashed line).

The late-stage subgroup is still distinct, compromising of the final two stages, though smaller, further corroborating the small difference inclusion of ADAS-Cog had on the disease progression sequence. For completeness, Table S1 provides a group-level comparison between the whole cohort and subgroups, where the most notable difference is in the difference between baseline ADAS-Cog scores in the early- and middle-stage subgroups.

Kaplan-Meier survival curves are shown in Figure S4 for the whole cohort (**A**), early-stage (**B**), middle-stage (**C**), and late-stage (**D**) subgroups. The late-stage subgroup is similar to that of the main paper (Figure 4**C**), despite the smaller size (92 vs. 121), which is perhaps unsurprising given the similarity between the model sequences with and without ADAS-Cog scores. The lower rate of progression to AD in the early-stage subgroup compared to the middle- and late-stage subgroups supports the validity of the data-driven disease progression sequence, though no subgroup shows a significant difference between placebo and treatment groups.

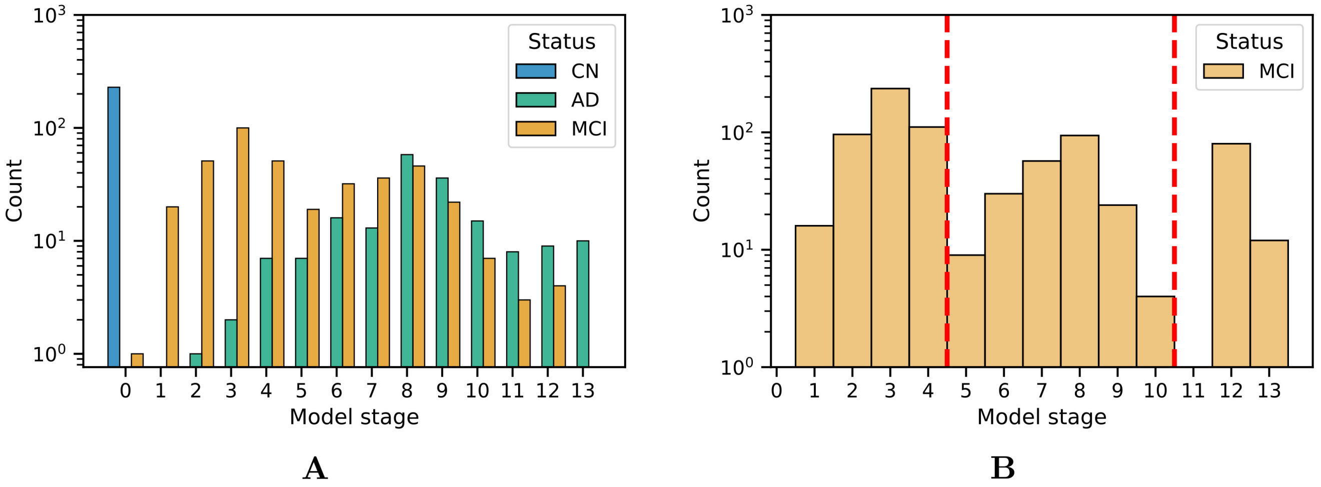

Figure S3. Histograms of model stage for subjects in the ADNI dataset (**A**) and ADCS-MCI trial (**B**).

Table S1. Demographic and Cognitive comparison of All ADCS-MCI trial participants and the model-determined subgroups.

	Group			
Measure	All (N=769)	Early-stage (N=648)	Middle-stage (N=121)	Late-stage (N=92)
Age (years)	72.9 (7.3)	72.3 (7.5)	74.3 (6.5)	72.7 (7.7)
Education (years)	14.6 (3.1)	14.9 (3.0)	13.8 (3.2)	15.1 (3.1)
Sex (% female)	352 (45.8%)	197 (42.9%)	111 (50.9%)	44 (47.8%)
APOE-e4 carrier (%)	424 (55.1%)	219 (47.7%)	146 (67.0%)	59 (64.1%)
Donepezil Arm (%)	253 (32.9%)	146 (31.8%)	81 (37.2%)	26 (28.3%)
Vitamin E Arm (%)	257 (33.4%)	152 (33.1%)	72 (33.0%)	33 (35.9%)
Placebo Arm (%)	259 (33.7%)	161 (35.1%)	65 (29.8%)	33 (35.9%)
ADAS-Cog 11	11.3 (4.4)	8.7 (2.9)	14.7 (3.6)	15.6 (3.2)
ADAS-Cog 13	17.7 (6.1)	13.8 (3.7)	23.3 (4.0)	23.9 (3.9)
ADAS-Cog Q4	6.3 (2.2)	5.0 (1.7)	8.3 (1.3)	8.0 (1.5)
Boston Naming	6.9 (2.4)	7.4 (2.2)	6.7 (2.3)	5.1 (2.6)
CDR Global	0.5 (0.0)	0.5 (0.0)	0.5 (0.0)	0.5 (0.0)
CDR Sum of Boxes	1.8 (0.8)	1.6 (0.7)	2.1 (0.8)	2.2 (0.8)
Clock Drawing	4.3 (0.9)	4.5 (0.8)	4.4 (1.0)	3.3 (1.0)
Digit Span bwd	6.2 (2.1)	6.4 (2.1)	6.2 (2.1)	5.1 (1.9)
DSST	31.5 (10.9)	34.7 (10.1)	29.4 (10.0)	20.4 (7.9)
Logical Memory - Delayed	3.3 (2.4)	4.3 (2.2)	1.8 (1.9)	1.8 (1.9)
Logical Memory - Immediate	6.2 (3.1)	7.4 (3.0)	4.8 (2.6)	4.3 (2.6)
MMSE	27.3 (1.8)	28.1 (1.6)	26.1 (1.4)	26.0 (1.7)
Verbal fluency - Animals	15.8 (5.2)	17.4 (5.2)	14.8 (4.1)	10.3 (3.1)

1.3 Summary

ADAS-Cog test scores are commonly-used as a primary endpoint in clinical trials as an assessment of multiple cognitive domains: episodic memory, language, and praxis (Kueper et al., 2018). Excluding it from our reference model (in the main paper) allowed secondary validation of the subgroups, but potentially limited the resolution of the staging that could be obtained. Although cognitive measures (in similar functional domains) will correlate, inclusion of ADAS-Cog into the EBM in these supplementary analyses

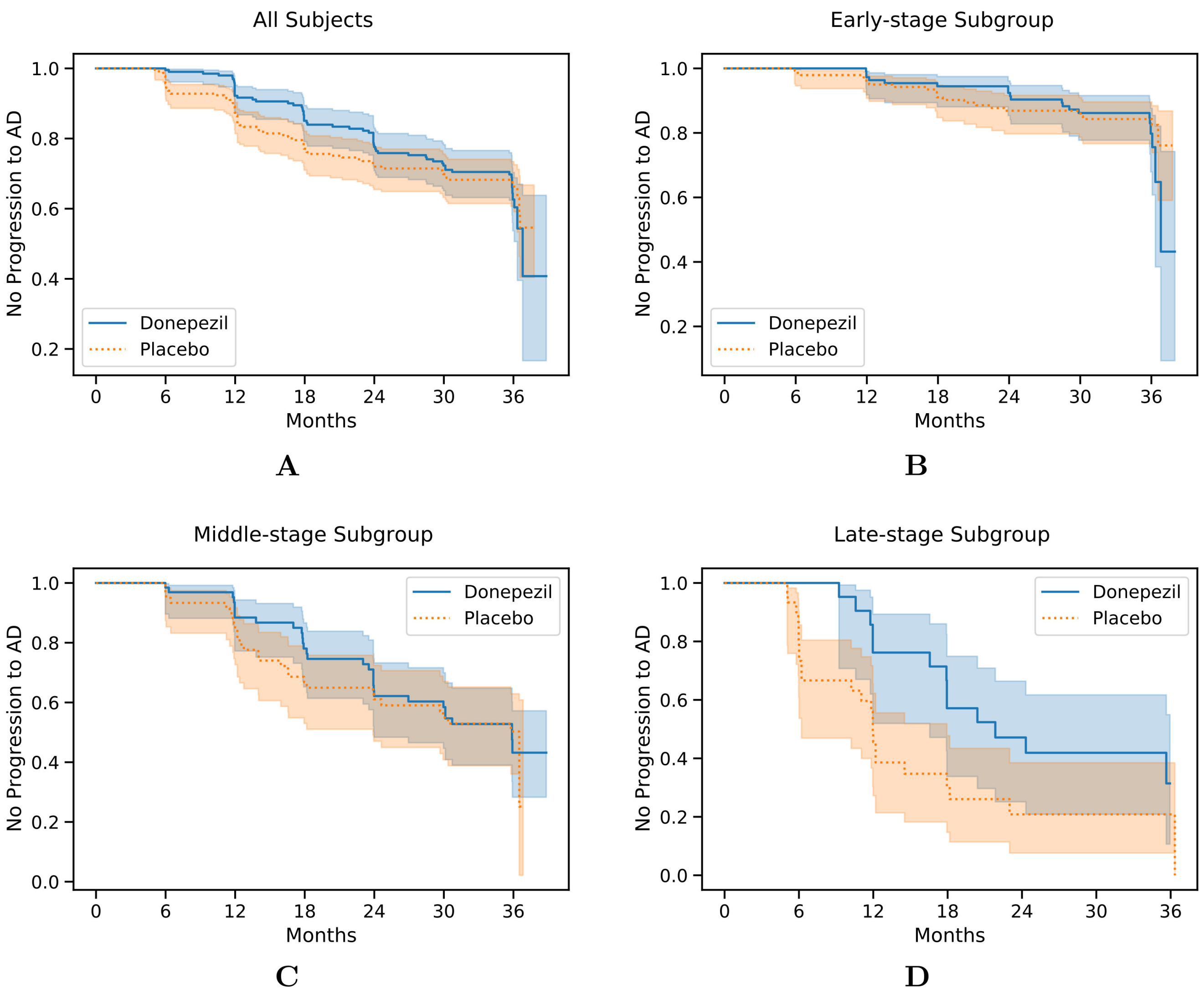

Figure S4. Kaplan-Meier survival curves for all 769 participants (**A**), the early-stage subgroup (**B**), the middle-stage subgroup (**C**), and the late-stage subgroup (**D**) in the ADCS-MCI trial.

has allowed further distinction between the early and middle stages to produce three subgroups. The observed hazard ratios indicate similar rates of progression to AD in the donepezil arm, suggesting that (in terms of the primary outcome of the trial) there is not a significant difference in the treatment effect in the early and middle stages of the identified disease progression sequence, despite a consdierable difference in the mean ADAS-Cog 13 at baseline (9.5 points).

Inclusion of ADAS-Cog into the EBM results in a greater separation of mean ADAS-Cog 13 throughout the trial between the treatment arms, though as this is not reflected in the primary outcome this just further supports the current use of donepezil for its cognitive benefits.

REFERENCES

Kueper JK, Speechley M, Montero-Odasso M. The Alzheimer's Disease Assessment Scale-Cognitive Subscale (ADAS-Cog): Modifications and Responsiveness in Pre-Dementia Populations. A Narrative Review. *J Alzheimers Dis* **63** (2018) 423–444.
